# Severe Euglycemic Diabetic Ketoacidosis Requiring Intubation After Tirzepatide and SGLT2 Inhibitor Coadministration in a Patient With Type 1 Diabetes Mellitus From a Large Tertiary Care Centre in Karachi, Pakistan: A Case Report and Brief Review of the Literature

**DOI:** 10.1002/ccr3.71929

**Published:** 2026-01-22

**Authors:** Maliha Malik, Hammad Amjad, Khadija Malik, Muddassir Syed Saleem, Shanzay Akhtar, Muslehuddin Paracha, Mobeen Abid, Nabahat Shafi, Ahmed Asad Raza, Abedin Samadi, Samar Abbas Jaffri

**Affiliations:** ^1^ Department of Medicine Liaquat National Hospital Karachi Pakistan; ^2^ Department of Medicine Karachi Medical and Dental College Karachi Pakistan; ^3^ Department of Medicine Civil Hospital Karachi Pakistan; ^4^ Department of Medicine Dow University of Health Sciences Karachi Pakistan; ^5^ Department of Medicine Jinnah Sindh Medical University Karachi Pakistan; ^6^ Department of Medicine Kabul University of Medical Sciences Abu Ali Sina Kabul Afghanistan

**Keywords:** case report, euglycemic diabetic ketoacidosis, incretin therapy, SGLT2 inhibitor, tirzepatide, type 1 diabetes mellitus

## Abstract

Euglycemic diabetic ketoacidosis (euDKA) is an uncommon but potentially life‐threatening complication that may arise in patients treated with incretin‐based therapies or Sodium–Glucose Cotransporter‐2 (SGLT2) inhibitors. We report a 41‐year‐old female with Type 1 Diabetes Mellitus (T1DM) who developed severe euDKA after initiating tirzepatide for weight loss while on empagliflozin and basal–bolus insulin therapy. She presented with severe vomiting and profound metabolic acidosis (pH 6.96, bicarbonate 1.5 mmol/L) despite only modest hyperglycemia (glucose 190–200 mg/dL). The severity of acidosis necessitated intubation and intravenous bicarbonate therapy. Laboratory findings revealed elevated amylase (688 U/L), suggesting possible tirzepatide‐associated pancreatic stress. No infection or other precipitating factor was identified. The patient recovered after intensive insulin and fluid replacement. This case highlights the risk of severe euDKA with the administration of tirzepatide in T1DM, particularly in combination with an SGLT2 inhibitor. Clinicians should keep a high index of suspicion for ketoacidosis in such patients despite normal or mildly elevated glucose levels and inform them of early detection of symptoms and sick day management.

## Key Clinical Message

Euglycemic diabetic ketoacidosis can occur in patients with T1DM receiving tirzepatide, particularly when combined with SGLT2 inhibitors. This case illustrates a severe presentation requiring intubation and bicarbonate therapy, underscoring the need for vigilant metabolic monitoring, early recognition, and cautious use of dual incretin therapy in insulin‐dependent patients.

## 1 Introduction

Euglycemic diabetic ketoacidosis (euDKA) is an uncommon but serious metabolic complication, defined by ketoacidosis (arterial pH < 7.3 or serum bicarbonate < 18 mmol/L) in the presence of normal or only mildly elevated plasma glucose levels, usually < 250 mg/dL (13.9 mmol/L) [1]. Although it represents only 2%–10% of diabetic ketoacidosis cases, the absence of significant hyperglycemia can obscure the diagnosis and delay treatment [2]. While euDKA has been most often linked to sodium–glucose cotransporter‐2 (SGLT2) inhibitors, other agents that affect insulin and glucagon balance have also been implicated [3, 4].

Tirzepatide, a dual agonist of glucose‐dependent insulinotropic polypeptide (GIP) and glucagon‐like peptide‐1 (GLP‐1) receptors, has been shown to produce marked improvements in glycemic control and weight reduction in type 2 diabetes [5]. Its off‐label use in people with Type 1 Diabetes Mellitus (T1DM) is increasing, particularly for weight management, but safety data in this group remain limited. Recent reports have raised concerns that incretin‐based therapies may contribute to ketoacidosis by reducing oral intake, altering glucagon suppression, or modifying insulin sensitivity [6, 7].

We describe a patient with T1DM on basal–bolus insulin who developed euDKA soon after starting tirzepatide for weight loss, in the presence of SGLT2 inhibitor use. This case adds to the emerging evidence on incretin‐related metabolic complications and highlights diagnostic and therapeutic considerations for clinicians.

## 2 Case History

A 41‐year‐old female with a history of T1DM since 2003 after a diagnosis of gestational diabetes presented to the emergency department with multiple episodes of non‐bilious, non‐projectile vomiting and severe nausea for several days. She reported no fever, abdominal pain, or dysuria.

Her diabetes was controlled with a glargine insulin regimen of 13 units daily and 8 units of insulin aspart prior to each meal. She also had been using empagliflozin 10 mg daily and recently started on tirzepatide 25 mg weekly for weight loss. Diet was not changed recently as well as no change in insulin dosage or any other medications. The patient initially presented with a body mass index (BMI) of 30 kg/m^2^ and continuous glucose monitoring revealed blood glucose of 120–200 mg/dL before presentation. Otherwise, there was no significant history.

## 3 Investigations, Diagnosis, and Treatment

According to the patient, symptoms began after the second dose of tirzepatide and were bad enough to make the patient unable to have meals. She was sent to the intensive care unit (ICU). On first presentation (day 0), arterial blood gas (ABG) analysis showed severe metabolic acidosis with pH 6.96, pCO_2_ 6.80 mmHg, pO_2_ 100 mmHg and serum bicarbonate 1.5 mmol/L. The results of the Laboratory test are summarized in (Table 1). The patient was placed on airway protection by intubation due to profound acidosis and empirically given intravenous insulin infusion, bicarbonate therapy, and broad‐spectrum antibiotics. Complete urine output was preserved during the admission.

**TABLE 1:** Laboratory results from initial presentation, admission and hospital stay.

Variable	Day 0 (initial presentation)	Day 0 (hospital ICU)	Day 1	Day 2	Day 3 (hospital ward)	Day 4	Day 5	Day 6 (discharge)
pH	6.90	7.16	7.33	7.46	7.46	7.43	7.49	N/A
pCO_2_ (mmHg)	6.80	11.70	23.20	39.99	39.90	31.10	29.00	N/A
pO_2_ (mmHg)	100	232	100	100	100	98	99	N/A
Bicarbonate (mmol/L)	1.50	8.30	14.40	27.10	27.10	21.50	23.70	22.00
Anion gap	N/A	N/A	25	19	19	N/A	18	N/A
Sodium (mmol/L)	133	143	148	147	144	141	141	143
Potassium (mmol/L)	4.6	5.2	3.3	2.9	3.8	3.4	3.1	2.9
Chloride (mmol/L)	100	106	108	109	106	103	101	102
Calcium (mmol/L)	N/A	0.89	0.67	0.76	0.77	0.90	0.85	N/A
Glucose (mmol/L)	N/A	13.7	15.4	10	8.8	N/A	N/A	N/A
HbA_1c_ (%)	N/A	7.03	7.03	N/A	N/A	N/A	N/A	N/A
Ketones	N/A	N/A	N/A	N/A	150	150	N/A	N/A
Lactate (mmol/L)	N/A	3.4	1.1	1.0	1.1	N/A	N/A	N/A
Hemoglobin (g/dL)	14.70	14.80	13.00	11.70	10.30	9.60	10.90	10.4
Platelets (×10^3^)	425	370	296	214	170	167	219	312
Total Leukocyte Count (cells/μL)	21.60	36.60	18.00	15.00	18.60	13.50	8.07	7.75

Abbreviation: N/A, Not Available.

ABG analysis repeated several hours later demonstrated pH 7.16, pCO_2_ 11.7 mmHg, pO_2_ 232 mmHg and serum bicarbonate 8.3 mmol/L. Urinary ketones were strongly positive (+4) and serum glucose levels were 190–200 mg/dL. These findings were consistent with euDKA. HbA1c was 7.03% and amylase levels were 688 U/L; whereas complete blood count, liver function tests and lipase levels were within normal limits. Abdominal ultrasound was unremarkable, and echocardiography demonstrated an ejection fraction of 55% without wall motion abnormalities.

The patient remained intubated and laboratory tests done after 24 h (day 1) showed improvement in acidosis. Blood sugar remained between 150 and 209 mg/dL, and urinary ketones were persistently positive at 150 mg/dL. The same management plan was followed, and insulin infusion was continued.

## 4 Results

On day 3, the patient significantly improved metabolically and was successfully weaned off the ventilator. Her leukocytes were temporarily elevated during recovery; they became normal prior to discharge. Blood, urine, and tracheal cultures showed no growth, so infection was not a trigger. She was sent to the ward after being clinically stable and discharged home on day 6 with a basal–bolus insulin regime and withdrawal of the tirzepatide. She was alert at discharge (GCS 15/15), hemodynamically stable, and oral intake tolerating.

## 5 Discussion

Although several cases of tirzepatide‐associated ketoacidosis have been reported internationally, this is the first documented case from Pakistan. This can be attributed to common underreporting of adverse events in low and middle‐income countries (LMICs) where pharmacovigilance systems are underdeveloped. Prescribing patterns and patient monitoring practices also differ regionally. By documenting this case, we expand the global safety profile of tirzepatide and raise awareness among clinicians who may encounter similar presentations [6, 8].

This case highlights the synergistic metabolic risks of coadministering tirzepatide and an SGLT2 inhibitor (empagliflozin) in patients with T1DM. Previous reports [7, 9, 10] involved overweight or obese female patients (BMI > 25 kg/m^2^) who were prescribed tirzepatide primarily for weight reduction and glycemic control similar to the reported patient. Initial pH of 6.96 and bicarbonate of 1.5 mmol/L indicated profound acidemia, which was more severe than most reported tirzepatide‐related euDKA cases and required intubation and aggressive sodium bicarbonate infusion. None of the previously published tirzepatide‐related euDKA cases required mechanical ventilation for respiratory compensation failure.

## 6 Literature Review

Literature review of published reports and case series indicates that severe complications such as diabetic ketoacidosis (DKA), euDKA, and pancreatitis, although rare compared to gastrointestinal side effects, are clinically significant. The overall incidence of acute pancreatitis in trials remains below 1%, yet some real‐world data indicate an elevated risk compared to non‐incretin therapies [11].

Across prior reports, patients typically presented with moderate metabolic acidosis, preserved or mildly reduced pH, and euglycemia. Louwagie et al. described a 35‐year‐old male with type 2 diabetes mellitus (T2DM) who developed DKA following tirzepatide and empagliflozin coadministration, with near‐normal glucose levels (89 mg/dL) [7]. Chaudhury et al. documented euDKA in a T2DM patient on tirzepatide monotherapy, showing that the drug alone can precipitate ketosis even without SGLT2 inhibitor use [9]. Furthermore, Sultan and Spencer reported *ketoalkalosis* after tirzepatide initiation, where alkalotic pH coexisted with high anion gap [10]. Sharma et al. reported DKA in a 36‐year‐old female with T1DM using a closed‐loop insulin infusion system and Continued Glucose Monitor (CGM) after starting tirzepatide for weight loss. Volume depletion from GI side effects led to CGM inaccuracy and insulin suspension, triggering DKA despite only moderate hyperglycemia. Both our case and the one reported by Sharma et al. highlight that tirzepatide‐induced dehydration can precipitate DKA via different pathways (metabolic or technological), emphasizing multifactorial risk with advanced diabetes therapies [12].

Compared with previously published cases, our case stands out for three reasons: concurrent T1DM and SGLT2 inhibitor use, the severity of acidemia requiring intubation and intravenous bicarbonate therapy, and biochemical evidence of pancreatic stress with markedly elevated amylase levels, suggesting possible tirzepatide‐related pancreatitis.

The pathophysiology of tirzepatide‐induced ketoacidosis is multifactorial. GLP‐1 receptor agonism leads to appetite suppression and delayed gastric emptying, resulting in reduced carbohydrate intake and starvation ketosis. Ketoacidosis has also been reported in nondiabetic patients receiving tirzepatide, where caloric restriction has been implicated as the major cause [13]. In diabetics, tirzepatide has been found to reduce the need for insulin, but once exogenous insulin delivery is not enough, relative insulin deficiency results in accelerated ketogenesis. Coadministration of SGLT2 inhibitors further enhances ketone production, increasing the risk of euDKA [7]. The overlap of these mechanisms most probably contributed to this severe presentation.

Emerging data suggest that genetic susceptibility may influence the risk of tirzepatide‐related metabolic complications. Polymorphisms in GLP‐1 and GIP receptors, as well as genes involved in ketone metabolism and glucose transport, may modulate individual responses and help explain why only a fraction of patients experience severe metabolic derangements [14, 15, 16].

## 7 Conclusion

This case underscores the need for the awareness of euDKA in patients on tirzepatide therapy, especially in association with SGLT2 inhibitors presenting with gastrointestinal symptoms, dehydration, or metabolic acidosis with normal glycemia. While proper diagnosis and care (e.g., ketone monitoring, temporary discontinuation in cases of vomiting/anorexia, and prompt medical attention) are recommended, this case highlights a need to increase scrutiny in the administration of pharmaceutical therapy in high‐risk patients, especially with high‐quality clinical evidence to guide stratification. Improvement of central adverse‐event reporting systems in LMICs like Pakistan, where the adoption of newer antidiabetic drugs is increasing, will be helpful in avoiding potentially life‐threatening reactions.

## 8 Limitations

Serum β‐hydroxybutyrate levels and pharmacogenomic tests were not conducted, and the documentation of the last tirzepatide dose timing was incomplete. Nonetheless, the detailed biochemical profile strongly supports the diagnosis of tirzepatide‐associated euDKA. Future research should focus on regional pharmacovigilance registries and mechanistic studies exploring genetic susceptibility to GLP‐1/GIP agonist–related metabolic complications [6, 8]. Targeted studies need to be carried out on obese diabetes patients to evaluate the safety and effectiveness of tirzepatide. Until potential safety information is accessible, tirzepatide should be administered with caution in diabetic patients. Our report adds to the scarce yet expanding literature on ketoacidosis related to incretin and emphasizes the necessity for additional systematic assessment.

## Author Contributions

**Maliha Malik:** project administration, supervision, writing – original draft, writing – review and editing. **Hammad Amjad:** conceptualization, data curation, project administration, supervision, visualization, writing – original draft, writing – review and editing. **Khadija Malik:** conceptualization, data curation, formal analysis, investigation, writing – original draft. **Muddassir Syed Saleem:** project administration, supervision, validation, writing – review and editing. **Shanzay Akhtar:** data curation, writing – original draft, writing – review and editing. **Muslehuddin Paracha:** investigation, writing – original draft. **Mobeen Abid:** data curation, writing – original draft, writing – review and editing. **Nabahat Shafi:** data curation, writing – original draft. **Ahmed Asad Raza:** writing – review and editing. **Abedin Samadi:** project administration, writing – original draft. **Samar Abbas Jaffri:** supervision, writing – review and editing.

## Funding

The authors have nothing to report.

## Ethics Statement

The authors have nothing to report.

## Consent

Written informed consent was obtained from the patient for publication of this case report. A copy of the written consent form is available for review by the editor‐in‐chief of this journal upon request.

## Conflicts of Interest

The authors declare no conflicts of interest.

## Data Availability

All data underlying the results are available as part of the article and no additional source data are required.
